# Postchikungunya Chronic Inflammatory Rheumatism

**DOI:** 10.1155/2016/7068901

**Published:** 2016-12-28

**Authors:** Keith A. Sacco, Razvan M. Chirila

**Affiliations:** Department of Internal Medicine, Mayo Clinic, Jacksonville, FL, USA

## Abstract

A 65-year-old male resident of Guatemala presented with a 5-month history of distal symmetric arthritis and generalized fatigue. This was associated with night sweats, chills, and weight loss. Symptoms were refractory to oral prednisone and hydroxychloroquine.

## 1. Introduction

Three clinical stages define the natural history of chikungunya infection: an acute stage (first 3 weeks), postacute stage (day 21 to end of the third month), and a chronic stage (after 3 months). Musculoskeletal signs and symptoms may be present throughout any stage of chikungunya infection. Presentation may vary between a self-limiting relapsing-remitting migratory arthritis to severe chronic inflammatory rheumatism in a minority of patients. Greater than 95% of patients suffer from postchikungunya musculoskeletal disorders (MSDs). The remaining minority develop a clinical inflammatory arthritis with synovitis that is frequently disabling and associated with a poor prognosis. The latter presentation is termed chronic inflammatory rheumatism (CIR) [1].

There has been a rise of chikungunya infection in South America together with a proportional rate of chronic inflammatory rheumatism. This highlights the need to diagnose such cases and aggressively treat patients deemed to have a poor prognosis from the outset of symptoms.

## 2. Case Presentation

A 65-year-old Chinese male resident of Guatemala presented with a 5-month history of distal symmetric arthritis and generalized fatigue. The patient described an insidious onset of pain, persistent stiffness, and joint swelling affecting the metacarpophalangeal and proximal interphalangeal joints, shoulders, knees, ankles, and feet in a symmetric fashion. This was associated with night sweats and chills but no fever. He endorsed weight loss yet denied any loss of appetite or change in diet. The patient initially self-administered oral acetaminophen and nonsteroidal anti-inflammatories with minimal improvement. Laboratory investigations of his constitutional symptoms were largely unremarkable. There was no anemia or leukocytosis on complete blood count with serum creatinine, serum electrolytes, and serum lactate dehydrogenase being within reference range. No bacterial growth was detected from blood cultures drawn. Further workup including colonoscopy and CT abdomen did not identify any other underlying etiology for his symptoms. He was evaluated by several rheumatologists with serology being negative for rheumatoid factor (RF). He was subsequently treated with low-dose prednisone with no improvement. The patient was maintained on oral hydroxychloroquine with no improvement as well.

Around two months prior to presentation in Guatemala, the patient had a fever up to 39°C associated with headache and neck and back pain which resolved spontaneously. This occurred soon after a mosquito bite. He had a concurrent transient skin rash over the medial thigh that resolved (around) three days after the mosquito bite. He was evaluated and investigated by an infectious disease physician and was found to be positive for chikungunya IgM.

On evaluation at our institution the patient had bilateral symmetric synovial swelling of the metacarpophalangeal and proximal interphalangeal joints together with mild wrist and ankle swelling with overlying warmth but no erythema. Joint pain was reproducible with knee flexion and radiocarpal joint mobilization in all directions of movement. Cardiorespiratory examination was otherwise benign and no focal neurologic deficits were elicited.

Laboratory studies showed microcytic anemia with a hemoglobin of 10.2 g/dL (13.5–17.5 g/dL), hematocrit 32.0% (38.8–50.0%), and low MCV of 65.9 fL (81.2–95.1 fL). His serum ferritin was high at 2732 mcg/L (24–336 mcg/L) with an erythrocyte sedimentation rate at 74 mm/hr (0–22 mm/hr) and C-reactive protein 138.5 mg/L (<7.0 mg/L). Immunologic testing was negative for rheumatoid factor, anticyclic citrullinated peptide (anti-CCP) antibodies, and antinuclear antibodies.

The patient's presentation was congruent with a symmetric inflammatory arthritis mimicking a clinical presentation of seronegative rheumatic arthritis.

## 3. Discussion

Given the prodromal phase between the acute febrile period and development of inflammatory arthritis following chikungunya exposure, the patient was diagnosed with postchikungunya chronic inflammatory rheumatism (CIR). Chikungunya is an arthropod-borne alphavirus endemic to West Africa; however outbreaks have been reported along the tropics around the Indian Ocean, Southeast Asia, and the Americas. It is transmitted among humans via the* Aedes* mosquito as its vector. It typically presents as an acute febrile illness following a 2–4-day incubation period. Headache, myalgia, gastrointestinal symptoms, and a distal arthritis are common associated symptoms that present with abrupt onset [2]. A maculopapular rash may emerge around 3 days following initial symptoms. Chikungunya IgM antibodies may be detected around five days from symptom onset with combined IgM and IgG antibodies being detected after ten days. Serum chikungunya IgG antibodies remain elevated thereafter due to acquired immunity to the virus [1]. Polymerase chain reaction assays can detect chikungunya RNA from symptom onset with higher sensitivity and specificity when compared to serology. Viral strain identification can also be performed [3].

Chronic arthralgia is a frequent persistent complication following chikungunya infection. A weighted model of pooled observational studies estimated a prevalence of 47.57% (95% CI 45.08–50.13), with a median time of onset estimated at 20.12 months from viral exposure [4]. However these musculoskeletal manifestations are widely heterogeneous. Arthritis, arthralgia, enthesopathy, and tendinitis have been commonly described [5]. The entity most commonly described is a self-limiting relapsing-remitting migratory arthritis of the ankles, wrist, and distal hand joints with synovitis. Retrospective data has shown complete resolution in 87.9%. However 5.9% were reported to develop a persistent painful deforming arthritis. These two facets of the disease may suggest separate pathophysiologic entities [6].

Postchikungunya CIR mimics rheumatoid arthritis in that patients develop a symmetric arthritis of the metacarpophalangeal (MCP), proximal interphalangeal (PIP), wrist, elbow, shoulder, knee, and metatarsophalangeal (MTP) joints. The median duration of morning stiffness is 90 minutes. DIP joint involvement was reported in 12.5%. Plain radiographs of affected joints show decreased joint space and periarticular osteopenia with erosions in about a third of patients. Synovial biopsy is negative for chikungunya and serology is generally negative for RF while anti-CCP antibodies are positive in half the cohort [7].

Supportive treatment including anti-inflammatory and analgesic medication is the mainstay of treatment for postchikungunya MSDs [8]. However this is not the case for postchikungunya CIR. It is suggested that persistence of arthritis after 3 months on corticosteroids underpins a diagnosis of postchikungunya CIR [1, 9]. Given that postchikungunya CIR mimics rheumatoid arthritis use of disease-modifying antirheumatic drug (DMARDs) has been advocated. Methotrexate is considered first-line therapy for postchikungunya CIR. High-dose corticosteroids and biological agents may have a role as second-line therapy. However, steroid-sparing agents should be used wherever possible due to steroid-related adverse effects [8]. Our patient was prescribed oral prednisone 40 mg for 2 weeks tapered to 30 mg in weeks 3-4 and 20 mg for a month thereafter. Oral methotrexate (weekly dose 15 mg) was coprescribed and consideration of another (DMARD) was envisioned if clinical responses were not observed. In a study of 16 patients with postchikungunya CIR combination therapy, hydroxychloroquine and sulfasalazine led to a positive clinical outcome in 12.5% after 3 months. Addition of methotrexate after 3 months in moderate and poor responders produced a good clinical response in 71.4% [6].

The rise of chikungunya infection in South America together with the high rate of chronic disabling inflammatory rheumatism highlights the need to stratify and aggressively treat patients deemed to have a poor prognosis from the outset of symptoms. Patients who present with RA-like seronegative inflammatory rheumatism would benefit from high-dose corticosteroids and DMARD combination to prevent permanent disabling joint deformity.

## References

[1] Simon F., Javelle E., Cabie A. (2014). Societe de pathologie infectieuse de langue, f. (2015). French guidelines for the management of chikungunya (acute and persistent presentations). *Médecine et Maladies Infectieuses*.

[2] Burt F. J., Rolph M. S., Rulli N. E., Mahalingam S., Heise M. T. (2012). Chikungunya: a re-emerging virus. *The Lancet*.

[3] Panning M., Grywna K., van Esbroeck M., Emmerich P., Drosten C. (2008). Chikungunya fever in travelers returning to Europe from the Indian Ocean Region, 2006. *Emerging Infectious Diseases*.

[4] Rodriguez-Morales A. J., Cardona-Ospina J. A., Villamil-Gómez W., Paniz-Mondolfi A. E. (2015). How many patients with post-chikungunya chronic inflammatory rheumatism can we expect in the new endemic areas of Latin America?. *Rheumatology International*.

[5] Schilte C., Staikovsky F., Couderc T. (2013). Chikungunya virus-associated long-term arthralgia: a 36-month prospective longitudinal study. *PLoS Neglected Tropical Diseases*.

[6] World Health Organization (2008). *Guidelines on Clinical Management of Chikungunya Fever*.

[7] Ganu M. A., Ganu A. S. (2011). Post-chikungunya chronic arthritis—our experience with dmards over two year follow up. *Journal of Association of Physicians of India*.

[8] Simon F., Parola P., Grandadam M. (2007). Chikungunya infection: an emerging rheumatism among travelers returned from Indian Ocean islands. Report of 47 cases. *Medicine*.

[9] Foissac M., Javelle E., Ray S., Guérin B., Simon F. (2015). Post-chikungunya rheumatoid arthritis, saint martin. *Emerging Infectious Diseases*.

